# Chemisorption and Reactions of Small Molecules on Small Gold Particles

**DOI:** 10.3390/molecules17021716

**Published:** 2012-02-09

**Authors:** Geoffrey C. Bond

**Affiliations:** Brunel University, Townfield, Rickmansworth WD3 7DD, UK; Email: geoffrey10bond@aol.com; Tel.: +44-1923-774-156

**Keywords:** catalysis, gold, chemisorptions, oxidation, hydrogenation, electrocatalysis

## Abstract

The activity of supported gold particles for a number of oxidations and hydrogenations starts to increase dramatically as the size falls below ~3 nm. This is accompanied by an increased propensity to chemisorption, especially of oxygen and hydrogen. The explanation for these phenomena has to be sought in kinetic analysis that connects catalytic activity with the strength and extent of chemisorption of the reactants, the latter depending on the electronic structure of the gold atoms constituting the active centre. Examination of the changes to the utilisation of electrons as particle size is decreased points to loss of metallic character at about 3 nm, as energy bands are replaced by levels, and a band gap appears. Detailed consideration of the Arrhenius parameters (E and ln A) for CO oxidation points clearly to a step-change in activity at the point where metallic character is lost, as opposed to there being a monotonic dependence of rate on a physical property such as the fraction of atoms at corners or edges of particles. The deplorable scarcity of kinetic information on other reactions makes extension of this analysis difficult, but non-metallic behaviour is an unavoidable property of very small gold particles, and therefore cannot be ignored when seeking to explain their exceptional activity.

## 1. Introduction

Growth in our knowledge of gold’s potential as a catalyst has increased rapidly since the discovery by Haruta and his colleagues of its remarkable ability to effect the oxidation of CO at moderate temperatures [1]. It is now also known to catalyse the water-gas shift, various hydrogenations, selective oxidations and reactions of environmental importance [2]. This ability is however largely confined to particles that are smaller than about 5 nm; they may be supported or be dispersed in a liquid medium as a colloidal suspension. Small gaseous clusters also perform reactions, but perhaps not catalytically. The hydrochlorination of ethyne to vinyl chloride catalysed by gold species gave an early stimulus to the study of gold catalysis [2], but the reaction does not illuminate the question of particle size dependence of rates. On moving to sizes below about 5 nm, activity often continues to increase ever more rapidly, but the reason for this is still a matter for debate, and it continues to be a fertile area for theoreticians [3]. This paper tries to cast a little further light on this interesting question.

Physical and chemical properties of gold particles undergo significant changes as their size is diminished ([Section 3), and the rise in activity has been attributed to many of them. Unfortunately this has often been done naively, because ‘activity’ depends on the extent of chemisorption of the reactants and on the form they adopt; basic kinetic theory then expresses the rate as a function of these variables, and establishing their dependence on physical structure and chemical composition of a gold catalyst is an essential prerequisite to understanding why activity varies as it does [4]. This task is however rendered all the more difficult by the lack of high-quality information on the reaction kinetics; many researchers feel it is adequate to characterise activity by a simple conversion vs. temperature plot, performed only once and in one direction. Studies reporting orders of reaction and activation energies are rare, notwithstanding their informative value. The commonly observed deactivation during use is a partial excuse effort this, but the main reason may be that those performing the work were never instructed in elementary kinetic theory , believing that merit accrues from simply finding a catalyst that is more active than others under some limited set of conditions. The likelihood of mass-transport limitation obtruding at high conversion and of non-isothermal conditions prevailing due to large heats of reaction is often overlooked [5].

When searching for possible causes for the size-dependence of activity, it is natural to look first at the changing surface/volume ratio, and consideration of static models of various crystal forms immediately suggests that the proportion of atoms of low coordination number occurring at edges and corners between plane areas increases quickly as size is lowered [4], and they are often therefore identified as the locus of the activity. Theoretical studies also indicate a different electronic structure for atoms of higher coordination number than those either on the plane surface or below [6]. For supported particles, e.g., those of hemispherical form, the fraction of atoms at the periphery and thus in touch with the support also increases, and such atoms are often allocated a specific role in catalysis. The following section touches on some other size-dependent alterations that may bear on catalytic acidity, but it needs to be stressed that *finding a correlation between ‘activity’ and any size-dependent factor does not of itself constitute an explanation of its cause*, which has to be traced through to the energetics of the transition state.

Previous studies based on the oxidation of CO on Au/TiO_2_ catalysts have led to the idea that a change in the electronic constitution of gold particles occurring at about 3 nm may be a principal cause of the high activity shown by particle smaller than this [4,7]. These studies raise certain other problems that will be explored further below; they did not for example address the question of how O_2_ comes to enter into reaction, as this is still a matter for debate. This review therefore centres on the evidence for the interaction of small molecules (CO, O_2_, H_2_, CO_2_, *etc.*) with small gold particles (<5 nm) as provided by studies of their chemisorption, and of their reactions with each other (e.g., CO oxidation, the water-gas shift, H_2_O_2_ synthesis), as well as reactions where di- or triatomic molecules are products (HCOOH decomposition). Except in the case of CO, which lends itself to easy study, information on their chemisorption is somewhat limited, and except for CO oxidation studies of the size-dependence of rates are almost totally lacking. Our task is therefore to trawl through the available literature to find hints and indications that small molecules in general respond favourably to the environment of very small gold particles, and hence to suggest where more systematic studies might usefully be performed. Observations made on massive gold (e.g., single crystal surfaces) and large particles are not considered, except to contrast them with those made on small particles; the many interesting features shown by bimetallic catalysts will likewise be passed over, as raising numerous problems that it would be premature to consider.

## 2. Chemisorption and Catalytic Activity

As noted in Section 1, any attempted correlation of the catalytic activity of a gold particles with its physical or chemical properties must necessarily be indirect, since activity is determined by the manner in which reactants and species derive from them are chemisorbed on the surface, that it to say, on the type of new chemical bonds that are formed. For the molecules we wish to consider, their chemisorption may be either associative (e.g., CO) or dissociative (e.g., H_2_, O_2_, H_2_O), and the new bonds’ strengths are an important variable, since strongly held species will tend to be unreactive, although they will cover the available sites extensively. In the simplest case where two different atoms A and B adsorb without dissociation on the same site, the rate *r* is given by:

*r* = *k* θ_A_θ_B_

where θ is the surface coverage and *k* the rate constant. This constant comprises two others that are revealed by the Arrhenius Equation when the temperature is changed, *viz*.:

*k* = *A* exp(−*E*/*RT*)

where *A* is the pre-exponential factor, *E* activation energy and *R* the gas constant [2,5]. The usefulness of this equation will appear later.

## 3. The Electronic Structure of Gold

When chemisorption occurs, new chemical bonds are formed between the atom or radical concerned and gold atoms on the surface; the strength of these bonds depends on the number and type of electrons that the gold can supply. To understand what happens with small particles, we must first examine the electronic structure of massive gold [2,4,8].

**Figure 1:**
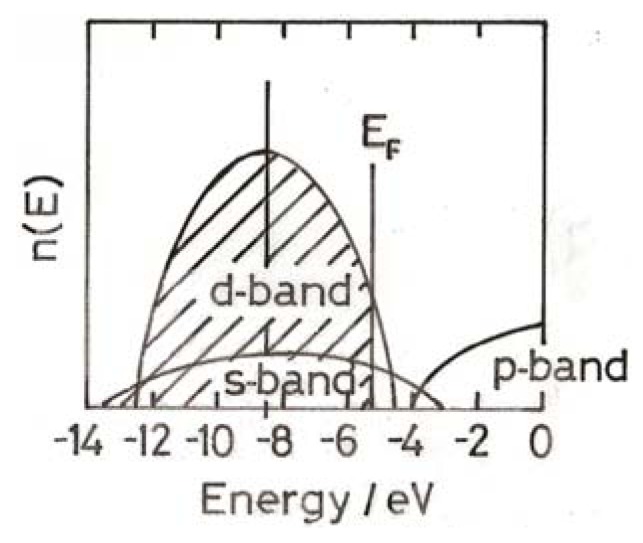
Distribution of energy levels for a large (20 nm) particle; note E_F_ lies below the top of the *d*-band, accounting for the existence of a small white line.

In a single atom, all electrons occupy distinct energy levels, but when two or more atoms come together their electrons must of necessity take slightly different energies, which together constitute an energy band. Thus in the case of gold we are concerned with 5*d*, 6*s* and 6*p* levels and bands, the electronic composition of the free atom being 5*d*^10^6*s*^1^. It is also important to know how the number of electrons varies within the energy band (the electron level density); the 5*d* band is quite narrow, and the overlapping 6*s* band much narrower (Figure 1). In the massive metal or large particles the 5*d* band is not quite filled, as indicated by a weak white line in the XAFS spectrum, and the highest occupied level (the HOMO), termed the Fermi level, therefore lies just below the top of the 5*d* band (Figure 1).

The chemical properties of gold are greatly influenced by the relativistic stabilisation of the 6*s*^2^ electron pair, which makes it keener to acquire an electron to form it as Au^−^ than to lose one to form Au^+^. This accounts for the extensive chemistry of the Au^−I^ state, for its high electronegativity (2.4 compared to 1.9 for Ag relative to Li = 1), and for its very high standard redox potential for Au^+^/Au^0^ of +1.691 V (+0.799 V for Ag^+^/Ag^0^) [9]. The first ionisation potential is also high (890 kJ mol^−1^ compared to 731 kJ mol^−1^ for Ag). These properties contribute to the lack of reactivity of plane gold surfaces in chemisorption and catalysis, and hence to its inability to corrode.

## 4. Electronic Structure of Small Gold Particles

The valence electrons of gold atoms in a particle are employed in one of two ways; some are used to form bonds to near neighbours, while for surface atoms some are surplus to requirements because they have less than 12 near neighbours. The way in which physical properties vary with size is largely determined by the changing fraction of surface atoms, and hence by the fraction of surplus electrons. They are responsible for surface energy (corresponding to surface tension in liquids), but their effect permeates the whole particle since interatomic distance decreases with size [10]. While it is sometimes convenient to consider geometric/structural parameters separately from optoelectronic factors, both find their origin in the way valence electrons are used.

**Figure 2:**
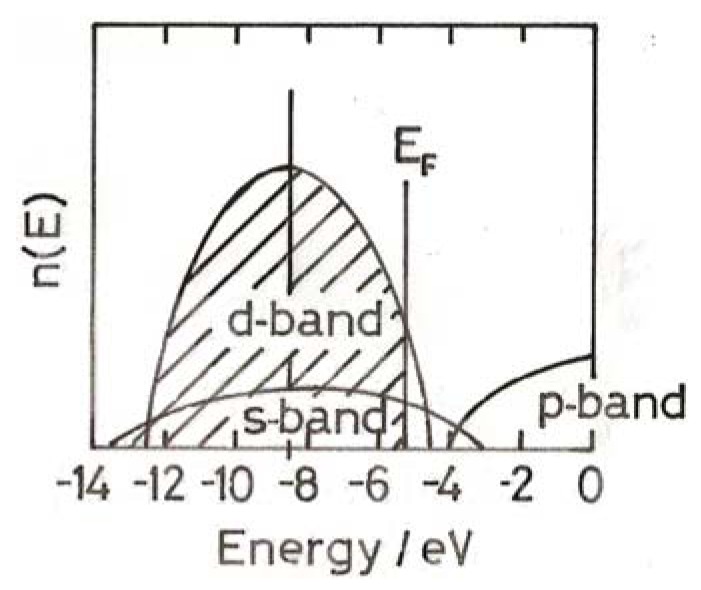
Distribution of energy levels for a very small (2 nm) particle, with a band gap above the Fermi level.

Information on the size-dependence of physical properties tells us how the use of valence electrons changes, and leads us towards a detailed description of the electronic structure of small gold particles. In order to establish what changes may occur, we have to examine the behaviour of optoelectronic properties, which include XPS (X-ray photoelectron spectroscopy) [11], STS (scanning-tunnelling spectroscopy) [12], ESR (electron-spin resonance) [13,14], and the optical absorption spectra (colours) of colloidal dispersions [15] or supported particles [14,16]. These techniques when applies to small gold particles disclose several important changes to the electronic structure. To keep things simple, we consider just a 2 nm particle [4]. Here the hybridised 5*d*6*s* band lies totally below the Fermi level, and the white line is weaker than for massive gold; there is a gap of about 1 eV (~96 kJ mol^−1^) between the Fermi level and the unoccupied 6*s*6*p* band (Figure 2) [4]. This particle is therefore non-metallic, unlike the massive metal and larger particles (Figure 1), but a semi-conductor and such particles ought therefore to behave as large molecules. In an alternative formulation, there is not a single band gap but a series of wide intervals between levels in a broad *sp* hybridised band that overlaps the 5*d* band; this model is suggested by the very large increase in photoluminescence quantum yield (×10^5^) in particles smaller than 2 nm compared to the value for massive gold [17].

The effects of these changes in electronic structure are to be seen in a number of other features shown by small gold particles. The 4*f*_7/2_ binding energy shown by XPS increases by about 1 eV at size <5 nm [11], and interatomic distance decreases [10]. Dramatic alterations to the colour of colloidal dispersions [8,15] were first remarked by Michael Faraday [18].

## 5. Chemisorption on Small Gold Particles

In considering the effect that these changes may have on the chemisorption of small molecules and on their subsequent reactions, we focus on three main situations: (i) atoms tightly packed in a flat surface of massive gold [e.g., Au(111)] having coordination number CN 9; (ii) those at the edges and corners of metallic particles (CN respectively 7 and 4 for an octahedron); and (iii) all surface atoms in very small particles, most of which will have CN between 3 and 7.

Atoms in plane surfaces are unreactive in chemisorption and catalysis essentially because they are reluctant to release electrons to form chemisorption bonds; such activity as there is appears to be confined largely to atoms around defects. Edge and corner atoms on particles have more ‘spare’ electrons those in planes, and DFT calculations suggest them to have a higher concentration of *d*-band vacancies than other atoms, and therefore a greater propensity for chemisorptions [6]. Each class of surface atom may have its own individual ‘density of states’ (*i.e.*, an electronic structure of the type shown in Figure 1 and Figure 2), each having its own band width and Fermi energy, but influenced by the structure of its neighbours. With very small non-metallic particles (<3 nm), most surface atoms are likely to participate in chemisorption; their electronic structures will either be similar to that in Figure 2, with a band gap, or have well-spaced energy levels in a *ds*-band [4].

There are two other factors that require consideration: The first is the question of the support. Unsupported particles (*i.e.*, colloidal dispersions) are catalytically active, but are suited only for reactions in the liquid phase. Very many types of support have been tried, but we will focus on oxides. Here we must distinguish between (i) ceramic oxides that are essentially non-reducible (SiO_2_, Al_2_O_3_); and (ii) oxides of transition metals (CeO_2_, TiO_2_, Fe_2_O_3_, *etc.*) where some degree of reduction is possible, the importance of which will depend on the way in which the catalyst is prepared.

Supported gold catalysts are prepared in two quite different ways. Much use is made of ‘model’ systems where gold atoms are deposited from the vapour phase onto single crystal oxide surfaces [e.g., Au/TiO_2_(110)] [2]. Particles are nucleated on reducible supports at anion defects created by the UHV conditions, which also dehydroxylate the surface. From these anion defects (F-centres containing trapped electrons [14]), which are particularly important in the case of ceria, some charge may be transferred to a nearby gold particle, raising the Fermi level and narrowing the *d*-band [14]; the charge may be concentrated on atoms at the interface and periphery, the particle then being described as being ‘electron-rich’. Such defects may also contribute to the catalytic process by providing an additional adsorption site [19,20].

Of greater utility are catalysts made by chemical deposition (e.g., by deposition-precipitation, DP) in aqueous medium [21]. Particles are then created on a stoichiometric surface coated with hydroxyl groups, and may seem positively charged if some gold cations remain at the interface at the end of the preparation. An equivalent view is that Au-O-Ti links will be formed in the process, the Au-O bond being polarised to give the gold a partial positive charge. Great care is however needed to assess the state of gold particles thus made, because preparation sometimes ends with an H_2_ reduction at quite high temperature (e.g., 573 K [14]), and this can form anion defects close to the particle by spillover of H atoms from the gold. This can counteract the effect just described, and the active gold sites at the periphery may behave as Au^δ−^, notwithstanding the continued presence of Au^δ+^ species at the interface. This will not happen if the preparation ends with a calcination, or if the catalyst is used with H_2_ or CO at too low a temperature for spillover to occur. The possible effects of variable ways of terminating the preparation are not however always recognised. There are strong indications, both experimental [22,23] and theoretical [24], that gold atoms at the periphery may be ineffective for adsorption and catalysis, perhaps for these reasons; with particles comprising *two* layers of atoms, activity is associated only with the *top* layer [25].

## 6. Chemisorption of Carbon Monoxide

The chemisorption of the CO molecule has been more intensively studied that that of any other molecule, largely because of the strength of IR absorption due to the vibration of the triple bond (ν = 1247 cm^−1^), and its sensitivity to the location of the molecule on the surface. With gold surfaces, absorption occurring at about 2110 cm^−1^ is always attributed to linear adsorption via the carbon atom at a single gold atom. There is no evidence that rehybridisation of the carbon atom from *sp*^2^ to *sp*^3^ and bridge-bonding to two different atoms, which occurs with other metals, especially palladium, is ever seen in the case of gold.

On metals possessing *d*-band vacancies, the bond is formed by forward donation of charge from the CO’s 5σ orbital, and back-donation of charge from the top of the *d*-band into the CO’s vacant 2π* antibonding orbital; this leads to a strong electroneutral C- metal bond together with some weakening of the C≡O bond. In the case of gold, the problem is to know which of these charge movements is dominant in each of the three sets of circumstances listed above [4,26]. In the carbonyl complex AuCl(CO) the C≡O frequency is 2162 cm^−1^, there being little or no π back-donation to the Au(I) as expected for the *d*^10^ configuration [27].

On the surface of massive gold, the chemisorption of CO is weak and only observable below ambient temperature [2]; work function change shows that charge moves *from* the CO *to* the metal, utilising the small amount of *d*-band vacancy. On small metallic particles (e.g., 3–4 nm in size [28]), the bonding is stronger, consistent with the appearance of greater activity for CO oxidation, and presumably employs mainly corner and edge sites. The steep rise in the rate of this process below ~3 nm (see Section 8) probably requires more extensive and more favourable CO chemisorption. Possible reasons for this have recently been discussed in detail [4]; in contrast to the situation with the massive surface, XANES measurements indicate charge transfer *to* the CO molecule, this being helped by a lowering of the Fermi level and a narrowing of the *d*-band. Theory [29] suggests that this results in an increase in the heat of adsorption (*i.e.*, of Au-CO bond strength) with decreasing size, and it was concluded [28] that both bonding mechanisms become more effective when the gold particle becomes non-metallic; a higher surface coverage by CO should result. In addition the presence of a band gap and increased energy level spacing may weaken repulsive interaction between the molecule and electrons in *sp* states.

## 7. Chemisorption of Oxygen

The chemisorption of O_2_ offers a great variety of possibilities. If it occurs on the metal, it may be associative (*i.e.*, molecular, with the O-O bond intact) or dissociative (*i.e.*, atomic, with two separate O atoms). The support if reducible may collaborate, using neighbouring anion defects (F centres containing trapped electrons), giving O_2_^−^ at the interface [3,19,23], or it may be formed using the gold’s 6*s*^1^ electron; complete transfer is not necessary, because a polarised Au…O_2_ bond may suffice. With ceramic supports, dissociative adsorption on the metal may be aided by OH species that have migrated from the support or, together with H atoms, have arisen from traces of water:

Au-OH → Au-O + Au-H

Au-H + O_2_ → Au-OOH → Au-O + Au-OH

The molecular form may be either sideways-on or linear; the atomic form may be either bridged or linear.

Chemisorption of O_2_ on clean massive surfaces does not occur, at least below 773 K, when an ordered atomic overlayer has been observed [30]; DFT calculations give activation energies of 150–215 kJ mol^−1^, which is enough to render the process very slow [2]. However when O atoms are provided by thermal or electrical excitation or via O_3_, they are quite strongly bonded, the Au-O dissociation energy by experiment and DFT calculation [31] being about 235 kJ mol^−1^. Compared to the O=O bond (497 kJ mol^−1^) it is however rather weak. The difficulty seems to arise in coaxing an electron from the gold to make O_2_^−^; its addition to the O_2_ 2π* orbital is needed to weaken the O=O bond prior to its dissociation, which requires only 408 kJ mol^−1^, but in view of gold’s electronegativity [9] it is not surprising that this step is hard. The O atom is however even more electronegative than gold, so that the Au-O bond will be polarized with the gold atom partially oxidized (Au^δ+^). Note that the dissociation energy of O_2_^+^ is higher than that of O_2_ (626 kJ mol^−1^), so that there is no advantage in removing charge to assist the bond-breaking.

Early attempts to observe the chemisorption of O_2_ on supported gold catalysts also found the process to be activated, but monolayer coverages were seen with ceramic supports above about 450 K, and tolerably good agreement with particle sizes measured by H_2_ titration and physical methods was obtained, assuming an O/Au ratio of 1:2 (*i.e.*, one O atom bridging two Au atoms) [2]. Estimated sizes ranged from 2 to 70 nm. The lower temperature at which extensive chemisorption occurred, compared to massive gold, signifies a role for low coordination number atoms in initiating the process; the need for elevated temperature may be connected to thermal excitation of the metal’s electrons and to increased kinetic energy of the arriving molecules [2]. Partial or complete release of electrons towards the O_2_ molecule is more likely at edges and corners where the work function is lower; this fact formed the basis of field-emission microscopy, a technique no longer in favour. With particles below 3 nm in size, on both reducible and ceramic supports, XANES measurements show considerable *d*-band depletion following exposure to O_2_ even at 298 K, up to 15% of the atoms becoming oxidized [32]. With ‘model’ Au/TiO_2_(111), O atoms are more strongly bonded to thin particles (2 atoms thick) than to thicker (6 atom thick) particles, activation energies for desorption being [33] respectively 190 and 139 kJ mol^−1^.

It is difficult to avoid the conclusion that the limited amount of low-temperature chemisorption takes place largely on particles so small as to be non-metallic. The lowering of the Fermi energy and narrowing of the *d*-band should facilitate charge transfer to the O_2_ molecule and assist its dissociation.

## 8. Oxidation of Carbon Monoxide

The matters addressed in the two preceding sections unite when we consider the oxidation of CO, a reaction for which gold is renowned [2,34]. It is much more active than for example platinum because it chemisorbs CO adequately, but not too strongly, and in the form of small particles has ways of securing ready access of O_2_ to the reaction site. Points of especial interest are (i) the way activity depends on particle size; (ii) the reasons for the size dependence; and (iii) the mechanisms by which the reaction occurs. The term is used in the plural, because it is unlikely that a single mechanism applies to all catalysts in all circumstances. A complete review of proposed mechanisms is not attempted, but a few general comments are offered; attention is concentrated on matters that illuminate the chemisorption of the reactants.

The reaction is generally believed to take place either entirely on the gold particle, or with the participation of adjacent support sites. Much emphasis has been placed on sites at the periphery of the particle, including gold atoms or ions and support cations and anion defects where they may exist. What is never considered is the ‘spillover’ of CO molecules from gold onto the support, followed by its oxidation by O^2−^ ions, and re-oxidation of the resulting defects by O_2_. This is in effect a Mars-van Krevelen mechanism, and was used to explain the greater activity for CO oxidation by Pd/SnO_2_ compared to Pd/SiO_2_ and SnO_2_ alone [35].

Activity begins to increase as size falls below about 5 nm. In fact it proves extraordinarily difficult to obtain comparable and reliable rate measurements because of: (i) the frequently observed deactivation with time-on-stream; and (ii) the use of different O_2_/CO ratios [4,34,36]. This ratio is only independent of conversion when the stoichiometric 2:1 ratio is used; with the commonly used 1% CO in air, the ratio approaches infinity as conversion nears 100%, and particularly under these circumstances mass-transport limitation begins to be felt above ~75% conversion; (iii) A further complication is the variety of units used to express the rate. It may be given as a turnover frequency (TOF, units, t^−1^) based either on the estimated mean number of surface gold atoms, derived for example from the mean size determined by TEM and assuming some particle shape, or on the number of peripheral gold atoms similarly derived. Both make guesses as to the number of active sites (a single atom may not be an active site), and it is usually sufficient to express it as the rate per unit mass of gold [e.g., mol CO (g_Au_ s)^−1^] [2].

**Figure 3:**
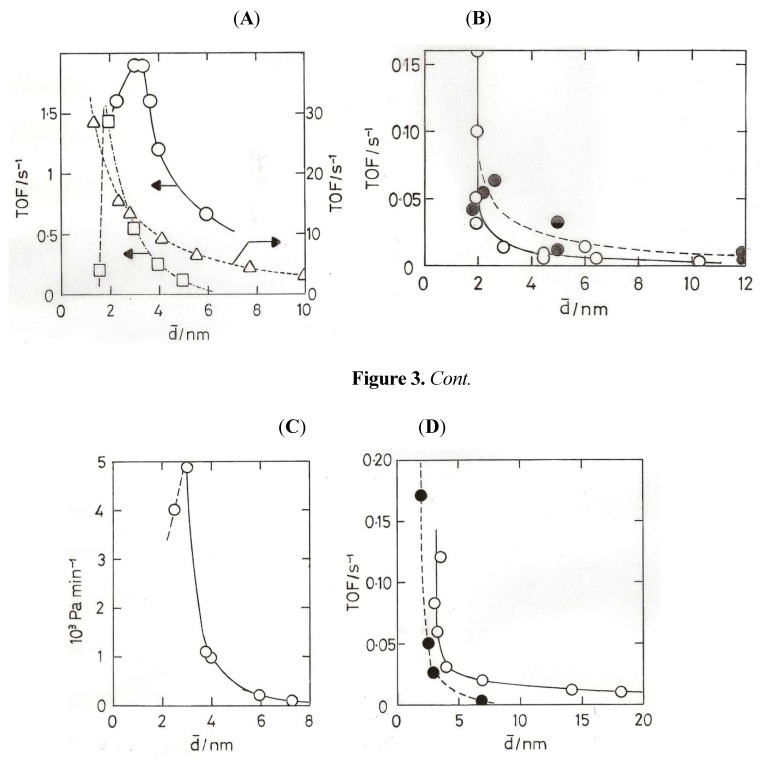
(**A**) ‘Model’ Au/TiO_2_(110) at 353 K [23]. (**B**) ‘Model’ Au/TiO_2_(110) at 473 K [37]. (**C**) ‘Model’ Au/TiO_2_(110) at 300 K [38]. (**D**) Au/TiO_2_ (open points) and Au/Fe_2_O_3_ + Au/Co_3_O_4_ (filled points) prepared by DP [41].

The increase of activity with decreasing particle size is observed with both ‘model’ systems using low area supports and methods to secure fairly narrow size distributions [23,37], and with chemically-made particles on higher area supports, where the range of sizes when measured by TEM is typically wider [4,38,39,40,41]. Within such a range there must also be a distribution of activities similar to that obtained from activity vs. mean size plots [4]. Both types of preparation give similar results between ~20 and 4 nm mean size, *i.e.*, in the range where most particles are expected to be metallic, the dependence of TOF on mean size being ~d^−3^, pointing to the importance of corner atoms as active centres. Examples are shown Figure 3A–D. Below ~4 nm there is a divergence of behavior: two studies using ‘model’ Au/TiO_2_ catalysts report maxima at either 3 nm [23] or 2 nm [37], although they are not found with chemically-prepared catalysts, where TOF frequently ‘lights-off’ at a mean size between 2 and 3.5 nm (see Figure 3B–D. At lower sizes, TOFs vary widely with little change in mean size, so that the exponent cannot be derived accurately. We must note however that the fraction of corner atoms also starts to rise rapidly below 3 nm, but this is based on assumptions concerning the shape and perfection of the particles. Early work using Al_2_O_3_ and SiO_2_ supports concluded that very active catalysts could not be made with them, but this was probably due to the difficulty of obtaining small enough particles. The development of suitable methods to get particles smaller than ~2 nm gave catalysts of acceptably high activity [34]; this confirms the idea (Section 7) that O_2_ chemisorption can occur on non-metallic gold particles even when the support cannot assist the process.

Analysis of the results obtained by Overbury *et al*. [39] has proved particularly informative. They used two Au/TiO_2_ catalysts (4.5 and 7.2% Au) calcined at a series of temperatures, and reported both TOFs and specific rates r (mol CO (g_Au_ s)^−1^) as well as apparent activation energies E (see Figure 3B for TOFs). It was therefore possible to calculate two sets of pre-exponential factors A_TOF_ and A_r_ using the Arrhenius Equation in the forms:

ln TOF = ln A_TOF_ − E/RT; ln r = lnA_r_ − E/RT

Now it is often found in heterogeneous catalysis that values of E and ln A are linearly related as:

ln A = m + cE

implying that a high E is compensated by a high ln A; perfect obedience to this compensation equation requires all rates to be equal at a unique isokinetic temperature T_i_ given by:

RT_i_ = c^−1^

**Figure 4:**
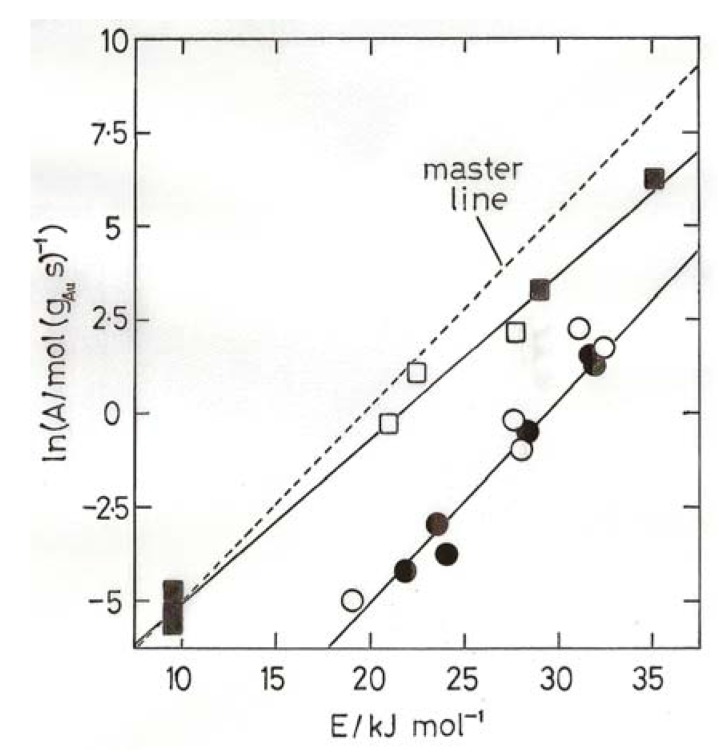
Compensation plot for CO oxidation over 4.5% Au/TiO_2_ (open points) and 7.2% Au/TiO_2_ (filled points) derived from ‘atomic rates’ (mol CO (g_Au_ s)^−1^) [39]. Square points, particle sizes, 1.8–2.6 nm; circles, 5–11.8 nm.

Using TOF values one obtained [4,7] a general sense of compensation but with points quite scattered; using specific rates however the points all fell around two well defined lines, one corresponding to higher activities and sizes below 3 nm, and another to lower activities and sizes above 3 nm (Figure 4). It has been suggested that points around a single line imply a similar mechanism, the apparent activation energies being moderated by the heats of adsorption of the reactants [4,42]. The abrupt change from one line to another has therefore been taken as a consequence of the change from metallic to non-metallic behavior of the gold particles, and the effect of this upon activity has been discussed in terms of the likely increase of CO bonding strength and coverage.

A wider inspection of the literature for Arrhenius parameters E and ln A revealed [4,7] a range of activation energies between about 8 and 70 kJ mol^−1^ lying about a single line close to but slightly steeper than the upper line in Figure 4; this ‘master’ line, also shown in Figure 4, is taken as a benchmark for catalysts of high activity. Its slope gives T_i_ = 234 K, so most of the data are collected above this temperature, where high values of E correspond to high activity. If this analysis is correct, it follows that all highly active catalysts adhering to the ‘master’ line will have gold in the non-metallic state.

**Figure 5:**
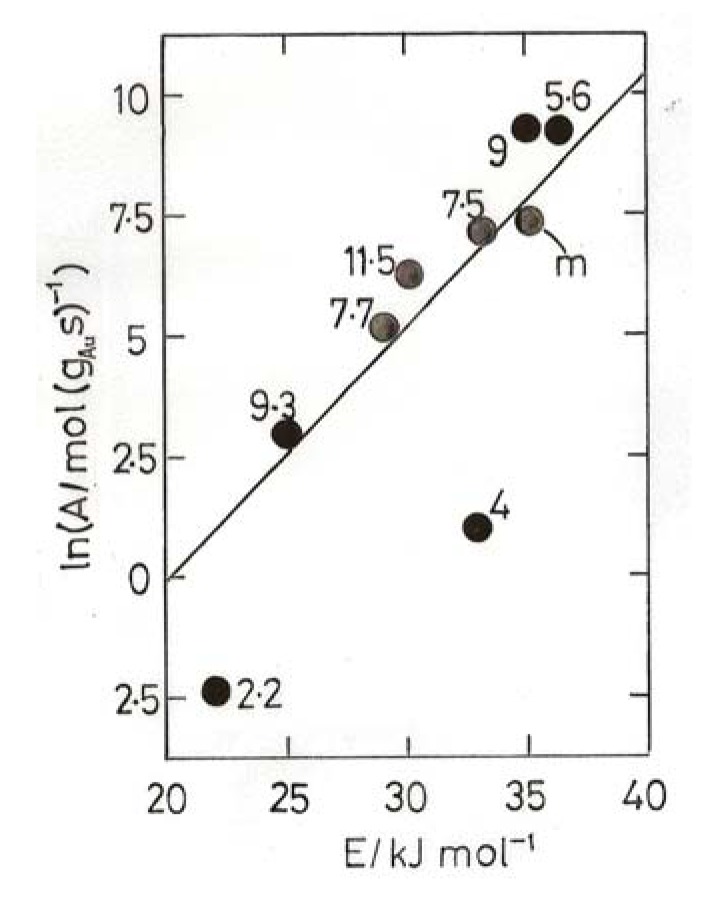
Compensation plot for CO oxidation over Au/TiO_2_ catalysts prepared by DP at various values of pH (indicated) [43]; the point m represents one made on a monolith support [44].

**Figure 6:**
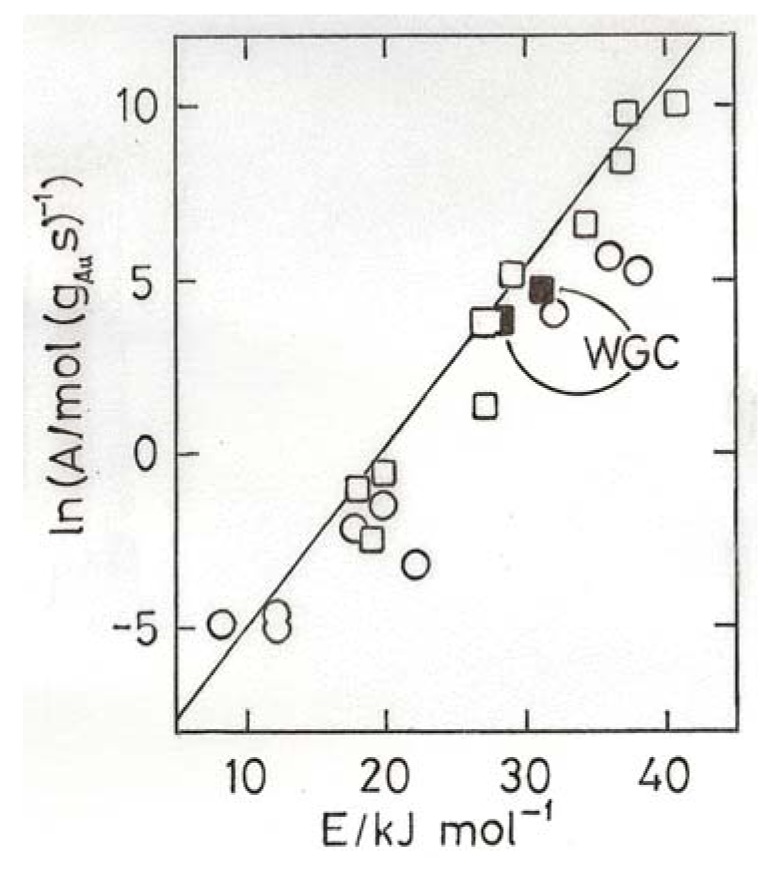
Compensation plot for CO oxidation over Au/TiO_2_ (squares) and Au/Al_2_O_3_ (circles) prepared by DP; based on results contained in references [2,12,45,46,47,48,49]. Two points for the World Gold Council reference Au/TiO_2_ catalyst are indicated.

**Figure 7:**
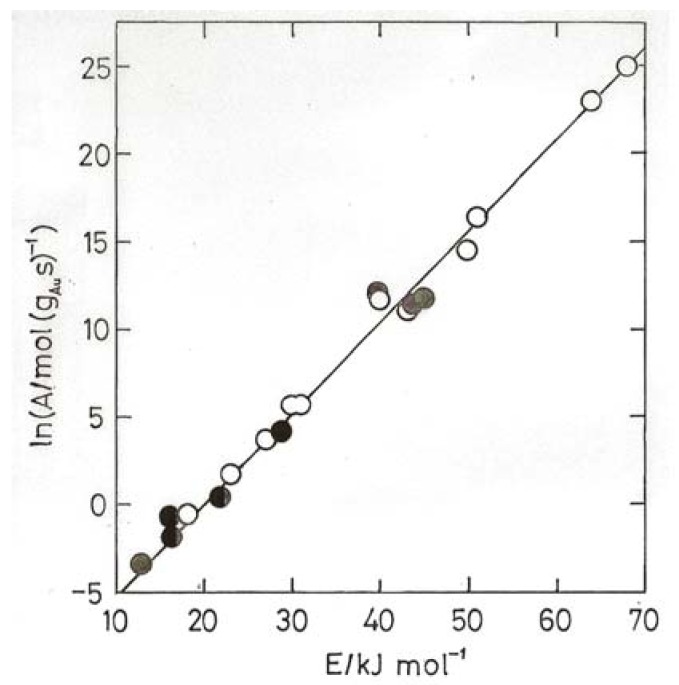
Compensation plot for CO oxidation over Au supported on mixed oxide supports (open circles, Fe + Sn, Ce, Zr, Ti [50]; Ce + La, Fe, Zr [51]) and single oxides (filled circles, Fe, Co, Ce, Zr, Si) [45,50,51,52].

The usefulness of compensation plots lies in their ability to display variations in activity irrespective of temperature providing activation energy and rate at a single temperature are known, whatever the temperature range used. Examples of this application are shown in Figure 4,Figure 5,Figure 6,Figure 7. From Figure 4 appears that the most active catalysts of Overbury *et al.* [39] are somewhat less active than the optimum. Figure 5 shows that Au/TiO_2_ catalysts made by DP at pH values of 5.6 and 11 have comparably good activity (above the benchmark line), while lower pH values give much less active products. 

Figure 6 gives results obtained by various groups for Au/TiO_2_ and Au/Al_2_O_3_ catalysts made by DP, including the World Gold Council’s Au/TiO_2_; all the points lying close to the benchmark line for Au/TiO_2_ have mean particles sizes below 3.5 nm. Clearly it is more difficult but not impossible to obtain good activity with alumina support. Finally in Figure 7 results are shown for catalysts made using Fe_2_O_3_, SiO_2_, Co_3_O_4_, ZrO_2_, CeO_2_ and a number of mixed oxides containing Fe_2_O_3_ or CeO_2_; catalysts having disappointing activities, mostly because of having large particle sizes, are omitted. The high activation energies shown by gold on mixed oxide supports containing Fe_2_O_3_ are especially noteworthy. Conventional kinetic analysis states that apparent activation energies E_a_ derived from rates will be lower than the true value E_t_ derived from the rate constant since the surface coverage will decrease with rising temperature, but to get true values requires measurement of the reaction kinetics at several temperatures. This has only been done once for CO oxidation, with a poorly dispersed Au/TiO_2_, but the clear conclusion was that the reactants were adsorbed non-competitively, *i.e.*, on different sites. The value of E_t_ was low (11 kJ mol^−1^). Determination of reaction kinetics is needed before the high activation energies shown in Figure 7 can be understood.

Evidently where E_a_ varies with particle size there is no temperature at which a unique form of dependence of rate on size can be found; it will change with the selected temperature. E_a_, derived from rate measurements, is a function of the heats of adsorption of the reactants and their orders of reaction [4,42]; the central question in such cases then becomes: what causes the variation in the heats of adsorption?

**Figure 8:**
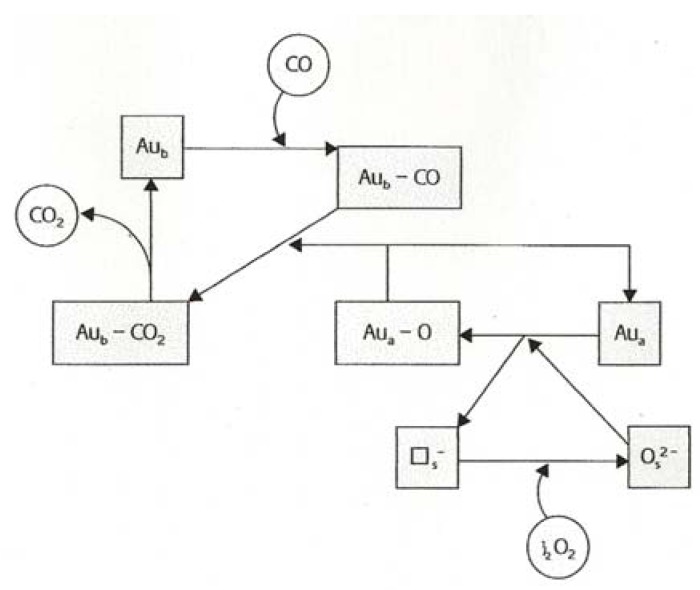
Example of a catalytic cycle: CO oxidation by the Mars-van Krevelen mechanism [34]. Encircled molecules are reactants and products; species shown in boxes are on the catalyst.

The subject of reaction kinetics leads us automatically to the question of reaction mechanisms. It has to be said that except for the work just mentioned [53] there is little information to go on. Mere measurement of orders of reaction expressed in Power Rate Law formalism (e.g., r = kP_CO_^x^P_O_^y^) gives at best a qualitative indication of how the reactants are adsorbed; a high order (~1) suggests weak adsorption, while a low order (~0) implies saturation of the relevant site, which may however form only part of the surface. Most of the proposed mechanisms are incomplete, and are based on informed speculation; standard kinetic analysis is usually ignored, being thought to lack photogenic appeal [5]. To begin with, there is no agreement as to what constitutes an acceptable statement of a mechanism; it may be a series of unit steps which when added give the stoichiometric reaction, or it may be depicted as a catalytic cycle (see for example Figure 8), but all too often attention is focused on the opening step. Identification of the rate-limiting step will lead to a prediction of the expected rate dependence on reactant and product concentrations, and any mechanism that does not match expectation with observation can only be classed as guesswork.

It is unfortunately necessary to admit the possibility that any mechanism will apply only to the conditions under which the measurements are made; thus differences in catalyst formulation and structure, and operating conditions, may all affect how the reaction proceeds. Among the issues that have been considered, but not fully resolved, are the oxidation state of the gold at the active site. Zero-valent gold is certainly more active than Au^3+^, which may however play a supporting role as in the Bond-Thompson mechanism [20], by anchoring OH groups at the periphery before they react with adsorbed CO; spectroscopic evidence for this has been obtained [26]. It needs to be established whether all unit steps proceed on the gold particle, as is likely when ceramic oxides are used as supports, or whether the support participates by for example allowing OH groups to migrate onto the gold, or with reducible oxides by employing anion defects close to the gold as sites for adsorbing O_2_ or O_2_^−^ [19,20]. There is evidence that associative chemisorption is possible on electron-rich particles [23], but in this form it only reacts with CO at 77 K after having been coerced to adsorb by the use of a supersonic RF-generated plasma jet [54]. It is formally possible for it to react without adsorbing, by a Rideal-Eley mechanism, but first-order in O_2_ is rarely observed. It may perform as –OOH formed by reaction of O_2_ with Au-H [55] or O_2_ or O_2_^−^ may react with –COOH created from CO + Au-OH or Au^x+^−OH [20]. If it dissociates, as it may well do on very small non-metallic particles (Section 7), O atoms will quickly be removed by CO. Power Rate Law kinetic statements show orders in both O_2_ and CO to range widely between zero and unity [2,20,56], but with very active catalysts the CO orders are equal to or <0.05, while O_2_ orders are 0.05–0.27 [41].

Water has a positive effect on rates, but poisons at high concentration [38]. It may serve to keep the support hydroxylated where this is important, and it certainly limits formation of toxic by-product species such as CO_3_^2−^ and HCO_3_^−^; but the way in which it acts has not been rigorously established, although suggestions have been made [20]. Water also plays a role in the selective oxidation of CO in excess H_2_, a system to be considered in Section 11.2.

One other unsolved probable deserves mention. It is sometimes seen that as temperature is lowered below about 220 K the reaction continues with a very small activation energy, thus allowing it to be observed to at least 185 K [57,58]. It is possible that the transition is due to a different form of O_2_ being stable at low temperature.

There are two other less known ways in which gold or gold compounds can catalyse CO oxidation, and which may throw indirect light on the normal catalytic mechanism:

(1) Au^I^Cl combines with CO on gentle warming to form the carbonyl Au^I^(CO)Cl as colourless, highly refractive tabular crystals [27]; it is also formed by passing CO over AuCl_3_. It reacts at once with water vapour [59]:
Au(CO)Cl + H_2_O → CO_2_ + AuCl + H_2_
(2) Gold atoms can be co-condensed with Ar, and they form a complex Au(O_2_) with O_2_, the molecule bonded sideways on; the complex reacts with CO to form the gold carbonyl peroxyformate which decomposes at 30–40 K to give AuO(CO) and then CO_2_ + Au [60].

## 9. Chemisorption of Hydrogen

Evidence for the chemisorption of H_2_ on gold comes from: (i) direct observations; and (ii) its ability to catalyse hydrogenations and reactions of its various forms, for which its dissociation is usually assumed to be necessary [61]. However the older literature on reactions catalysed by other Group 8–10 metals sometimes invokes the Rideal-Eley mechanism, by which the H_2_ molecule reacts with other adsorbed species without first dissociating.

Chemisorption of the H_2_ molecule does not occur on massive gold or large particles within the temperature range (300–500 K) of principal interest to catalysis [2]. Some adsorption does occur at sub-ambient temperatures, but even H atoms desorb at ~220 K, so that gold efficiently catalyses H atom recombination [62].

It has only quite recently been appreciated that H_2_ can in fact chemisorb on supported gold particles; this has been detected volumetrically and by XAFS/XANES [62,63]. With Au/Al_2_O_3_, adsorption isotherms reached plateaux at about 5 kPa pressure, the adsorbed quantity increasing slightly with temperature (300–473 K), suggesting that the process is activated. In the size range 1–3 nm it tended to increase as size diminished, but the results were somewhat scattered. The highest H/Au ratio was 0.73 at ~1 nm, and it seemed that adsorption was limited to edges and corners, with no migration to other sites. In XANES with Au/Al_2_O_3_ (1.3 nm), H_2_ adsorption caused the appearance of a small white line, indicating withdrawal of charge from the *d*-level pertaining to the sites involved; thus the adsorbed H atom had a slight negative charge. It therefore appears that, as with O_2_, chemisorption only occurs on particles below about 3 nm in size, *i.e.*, on those that are non-metallic [64,65]. Recent DFT calculations on Au/Fe_2_O_3_ suggest that low coordination number is needed, and that O atoms of the support appear to assist the process [66]. We must however remember that observing for example H/Au = 0.73 does not necessarily mean that particles of all sizes adsorb the same amount; the smaller ones might adsorb more and the larger ones less [4]. O atoms formed on Au/Al_2_O_3_ by chemisorption of O_2_ at 473 K have been titrated with H_2_, the amount consumed leading to size estimates in good agreement with TEM values [2].

## 10. Hydrogenation

### 10.1. Reactions Involving H_2_ and D_2_

The ready availability of two isotopes having essentially the same chemical properties permits the observation of processes that would otherwise be invisible. The equilibration of mixtures of H_2_ and D_2_ as:

H_2_ + D_2_ = 2HD ……K =~4

demands the dissociation of at least one, and more probably both reactant molecules. However with supported metal catalysts where the support S is coated with OH groups, the dissociation of D_2_ may be followed by spillover of the D atoms, and their replacing the H atoms of the OH groups:

D-Au + S-OH → Au + S-DHO → Au-H + S-OD

Thus any change in the overall H/D ratio implies the occurrence of this process; it has been found to happen with Au/SiO_2_ [67] and Au/Al_2_O_3_ [68].

**Figure 9:**
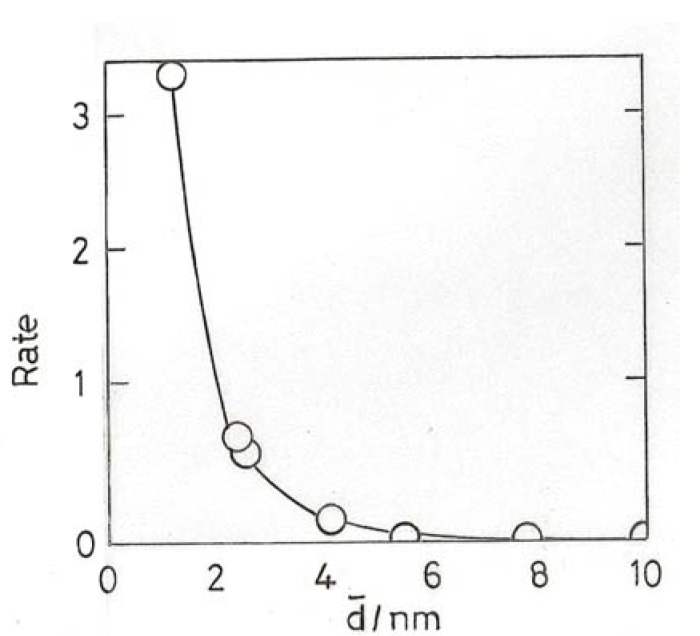
Rate of H_2_ + D_2_ equilibration (10^18^ molecules s^−1^) on Au/TiO_2_(110) at 425 K [24].

Equilibration has been observed on gold foil at high temperatures ((385–673 K) with large activation energies (90–115 kJ mol^−1^); presumably thermal activation of *d*-level electrons allows some dissociative adsorption to take place [69]. It also occurs with Au/Al_2_O_3_ at 298 K where gold particles are <3 nm in size; large particles require higher temperatures [61,70]. With Au/TiO_2_ catalysts, it was concluded by the use of FTIR of adsorbed CO and of DFT calculations to identify the active sites for H_2_ + D_2_ equilibration that adsorption was confined to edge and corner atoms not adjacent to the support [25], the existence of an Au-O-Ti link, which gives the gold atoms some positive charge, appears to inactivate it for chemisorption, a neutral atom able to donate negative charge to the molecule in order to weaken the H-H bond being needed. Measurements made with ‘model’ Au/TiO_2_(110) catalysts also point to a rapidly increasing rate below 2 nm (Figure 9) [24], it being dependent on the length of the periphery, although not necessarily that of the first layer of atoms. The activation energy was constant at 36.4 kJ mol^−1^, suggesting that the electronic structure of the active centre was always the same; this may be because only those particles smaller than ~3 nm, and therefore non-metallic, are active [4].

Questions concerning the exact mechanism of the equilibration remain. It would appear that H and D atoms are adsorbed on *single* gold atoms; reaction to form HD therefore needs *four adjacent* suitable gold atoms, surface migration being energetically prohibited. The only alternative is to invoke the Rideal-Eley mechanism [62], *i.e.*,:

Au-H + D_2_ → Au…H…D…D → Au-D + HD

for which only a single gold atom is required.

### 10.2. Hydrogenation of Unsaturated Hydrocarbons

The early patent literature reveals [71] a number of reports of uncertain reliability of gold’s ability to catalyse the hydrogenation of unsaturated hydrocarbons. The first detailed studies were reported in the 1970s; Au/Al_2_O_3_ and Au/SiO_2_ were found to hydrogenate ethene, 1-pentene, 1,3-butadiene and 2-butyne at quite modest temperatures [68]. This work also provided the first clear indication that (with Au/SiO_2_ at least) activity increased with decreasing particle size [13].

Subsequent work has understandably focused on the multiply-unsaturated hydrocarbons because of the industrial importance of their selective removal from alkene streams before their further processing. Palladium-containing catalysts while much more active than gold catalysts are nevertheless not perfect, particularly with regard to the formation of unwanted oligomers [72]. Several useful reviews of gold-catalysed hydrogenation have been published [61,70,73,74]. It is consistently found with 1,3-butadiene [75,76,77], ethyne [78,79,80] and similar compounds [73] that reaction stops at the mono-alkene stage, even in the presence of excess alkene [76]. Rates of hydrogenation of alkenes are much slower than for the diene [76] and alkyne [80], which are typically studied between 400 and 550 K. Some degree of deactivation caused by formation of strongly-held hydrocarbon residues is often observed [73,80].

**Figure 10:**
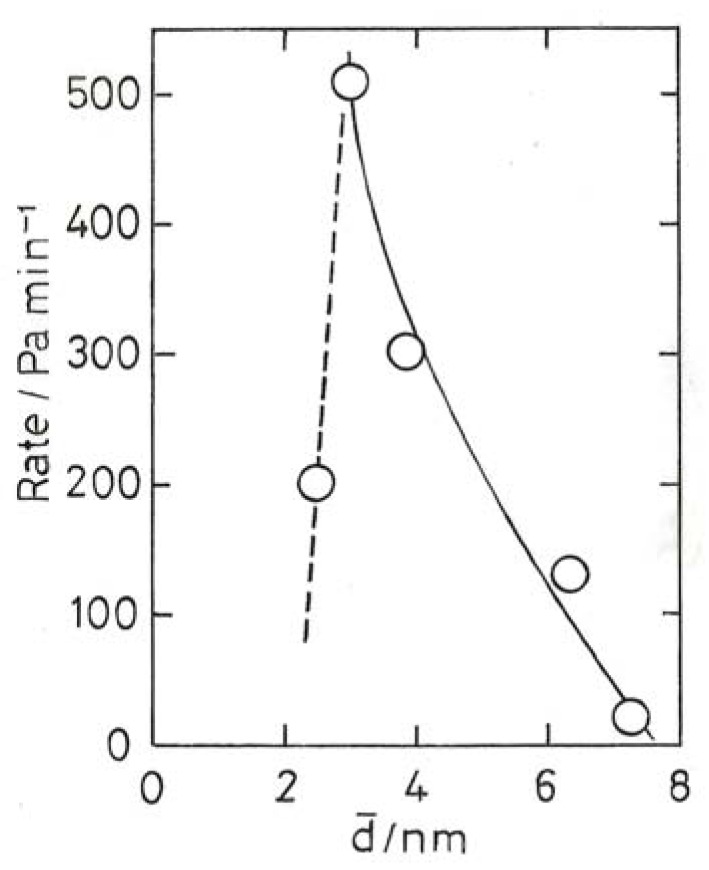
Rate of ethyne hydrogenation over Au/Al_2_O_3_ at 523 K [40].

When seeking information that relates to particle size effects, it is necessary to look not only for rates, but also for product distributions in the case of butadiene, and for kinetics (reaction orders *etc.*). In view of the very different conditions used (particle size, gold loading, support, temperature *etc.*), it is hardly surprising that no very clear picture emerges. Good rates have been found for ethyne hydrogenation with 2 nm Au/CeO_2_ [80] and <3 nm Au/Al_2_O_3_ promoted by Ce^3+^ [79]; rate maxima at ~3 nm have also been seen [40,73] (Figure 10), suggesting that a minimum size is needed. With the Au/CeO_2_, orders of reaction for ethyne and H_2_ are respectively −1 and +1 [79], implying that ethyne occupies the active sites to the virtual exclusion of H_2_, but with Au/Al_2_O_3_ they are respectively 0.1 and 0.4 [40], so that both reactants adsorb, the ethyne more strongly. Apparent activation energies between 30 and 37 kJ mol^−1^ have been reported [40,79,80]. Hydrogenation of 2-butyne gave 80% of *Z*-2-butene [69].

With 1,3-butadiene hydrogenation, rates were stated not to depend on particle size or support, but treating Au/CeO_2_ with CN^−^ solution to remove larger particles gave a catalyst of high activity [81]; Au^3+^ ions appear to resist dissolution, but may become reduced to give small particles during reaction. Reported relative amounts of the three possible pentene products (*E*-, *Z*- and 1−) vary considerably, with 1-pentene being always the major component [76,77,78]. Orders of reaction measured at 303–363 K were 0.7 for H_2_ and 0.2 for butadiene, independent of temperature; excess propene slightly inhibited (order −0.4) [69].

Gold-catalysed hydrogenation of alkenes has also provided results of interest. The reaction of ethene + D_2_ over Au/SiO_2_ gave deuterated ethenes and ethanes in amounts similar to those given by platinum catalysts, but very different from those afforded by palladium catalysts [13]. On small SiO_2_-supported particles, 1-pentene + H_2_ gave only *n*-pentane, its isomerisation to 2-pentenes only being seen with larger (5 nm) particles. It appears that alkyl reversal (*i.e.*, 2-pentyl → 2-pentene + H) is impossible on very small particles, perhaps due to lack of vacant sites to take the released H atom, molecular H_2_ being strongly adsorbed.

There is an almost total absence of information on the chemisorption of unsaturated hydrocarbons on small gold particles. It is for example not clear whether it is confined to the edge and corner sites presumed to be favored by H atoms, although the limited knowledge of reaction orders suggests there can be competition between H_2_ and the hydrocarbons.

Further information on gold-catalysed hydrogenation of other substrates (e.g., cyclohexene and 1,5-hexadiene) can be found in references contained in the cited reviews [61,73,74,75,76,77].

### 10.3. Chemoselective Hydrogenation

**Figure 11:**
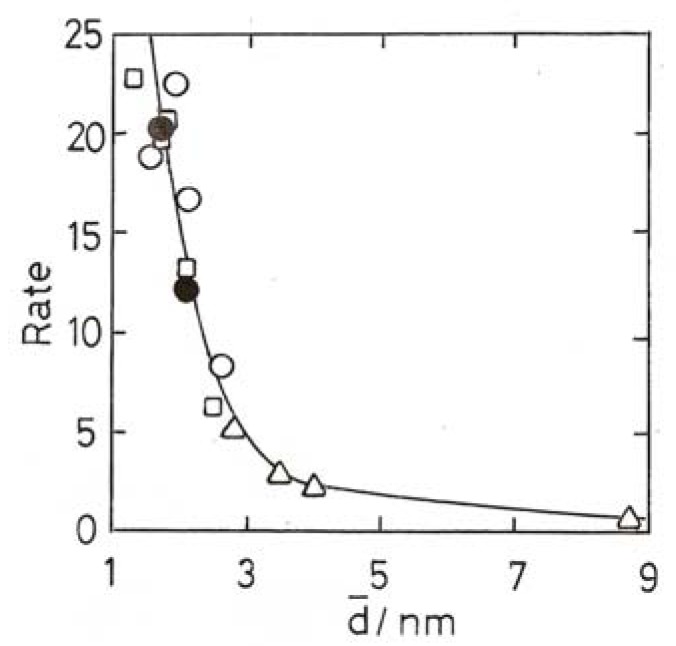
Rates of crotonaldehyde hydrogenation (10^−6^ mol (g_Au_ s)^−1^) over variously prepared and pretreated Au/TiO_2_ catalysts [83].

The term is applied when a molecule contains two or more reducible functions, only one of which it is desired to hydrogenate to obtain a more valuable product. Much attention has been given to molecules of the form R–CH=CH–CHO (R = H, acrolein (propenal), R = CH_3_–, crotonaldehyde, R = C_6_H_5_–, cinnamaldehyde) where the target is the unsaturated alcohol. Metals of Groups 8–10, unless modified, give mainly the saturated aldehydes and alcohol, but gold catalysts have given quite high selectivities (40%–60%) to the wanted products [14,75,77,78,82,83]. Severe deactivation is experienced, but selectivity is not affected by this or particle size or support [78]. The origin of the preferential reduction of the C=C function has been discussed in detail [14,83], and appears to be related to the ease with which CO is adsorbed on small particles. However the dissociative adsorption of H_2_ is probably rate-limiting; detailed results for Au/TiO_2_ catalysts [83] show a marked rate increase for smaller sizes (Figure 11) with a suspicion of a maximum at about 2 nm. At 513 K, Au/TiO_2_ catalysts are much more active (×10) than Au/ZrO_2_ catalysts [14], but due to a large difference in activation energies this factor is close to unity at 453 K. This emphasizes the importance of not relying solely on rates at a single temperature as a basis for comparing activities.

Gold supported on either CeO_2_, TiO_2_ or Fe_2_O_3_ effectively catalyses liquid-phase reduction of nitro-compounds at ~350–400 K, even in the presence of other reducible substituents (e.g., –CH=CH_2_) [84]. 1-Nitro-1-cyclohexene is hydrogenated to cyclohexanone oxime [85]. An unusually detailed kinetic study designed to identify the rate-limiting step for the reduction of nitrobenzene on Au/TiO_2_ showed this to be the dissociative adsorption of H_2_, the rate being proportional to its pressure and passing through a maximum as the nitrobenzene concentration was raised [86]. Temperature variation (343–423 K) of the reaction orders enabled the true activation energy to be determined as 31 kJ mol^−1^ from the rate constants; this value is close to those found for H_2_ + D_2_ equilibration (Section 10.1) and other hydrogenations (see above). Chemoselective reduction was believed to result from the adsorption of the –NO_2_ group either on the support adjacent to the metal or at the periphery, with the aromatic ring leaning against the higher part of the metal particle; substituents were thus unable to come in contact with the metal and therefore escaped hydrogenation [86].

## 11. Other Reactions of Small Molecules

### 11.1. Selective Oxidation of Carbon Monoxide in Hydrogen (PROX) and Oxidation of Hydrogen

The chemical industry needs a large amount of pure H_2_; this traditionally came via coal combustion to CO + CO_2_ and the Water-Gas Shift (Section 11.2):

CO + H_2_O → CO_2_ + H_2_

Latterly the steam-reforming of alkanes and alcohols has produced H_2_ contaminated with CO and CO_2_. The preferred method of removing traces of CO (CO_2_ is easily washed out) is by its selective oxidation to CO_2_ (PROX = preferential oxidation). One particular requirement that has attracted much attention is the need for CO-free H_2_ in proton-exchange membrane (PEM) fuel cells; the ‘idealised reformate’ (1% each of CO and O_2_ + 75% H_2_, balance inert gas) is commonly used for catalyst testing. A successful catalyst must remove CO to <10 ppm and less than 0.5% H_2_; the O_2_ selectivity (the fraction used to oxidize CO) should exceed 50% when CO conversion is >99.5%. The activation energy for H_2_ oxidation is higher than that for CO oxidation, so high selectivity is only obtainable at *low* temperatures. Conditions under which the Reverse Water-Gas Shift might occur must be avoided [2].

The possible simultaneous adsorption of three reactants, together with the effect of the product water on CO oxidation, makes for a quite complex system to study. The reducible oxides (TiO_2_, CeO_2_, Fe_2_O_3_) are the best supports, and Au/Fe_2_O_3_ calcined at 673 and than at 823 K just meets the above criteria [87]. A detailed kinetic study [26] that also reviewed earlier work led to the following conclusions. (1) H_2_ and CO compete for the same sites on gold particles, resulting in CO orders of reaction that are higher when H_2_ is present; orders in O_2_ are lower (~0.2–0.4) than those for CO; (2) The presence of H_2_ lowers the rate of CO oxidation; (3) Water counteracts the formation of species such as CO_3_^2−^ and HCO_3_^−^ that poison CO oxidation, but do not enter the main reaction pathway. Once again, high activities are only found with gold particles <3 nm in size [26].

There have also been a number of studies of the oxidation of H_2_ to water. A kinetic study [88] using Au/SiO_2_ and Au/MFI (a Ti-containing silicalite) showed qualitatively that rates increased as size diminished, but size distributions for the Au/SiO_2_ were broad; however, the similar orders of reaction (H_2_, ~0.7–0.8; O_2_, ~0.1–0.2) and the almost constant activation energy (~39 kJ mol^−1^) indicated that in all cases it was the same small size of particle that was active. Kinetic analysis pointed to two types of active centre; one adsorbed both reactants to give the HOO- radical, which then reacted with H_2_ to from H_2_O_2_ + H. The peroxide rapidly decomposed to water and O; the second site adsorbed only H_2_.

A great deal of effort has been devoted to the synthesis of H_2_O_2_ by direct oxidation of H_2_ [2,89]. It seems likely that the selective reaction proceeds by sequential reaction of an O_2_ molecule with adsorbed H atoms, giving first the HO2^.^ radical; it is improbable that the O_2_ molecule dissociates before the product H_2_O_2_ is formed, but in the absence of kinetic information the mechanism remains speculative. Most success has however been achieved with PdAu alloys, where the palladium speeds up the supply of H atoms to the active centre; platinum addition has the same beneficial effect on other hydrogenations [86]. None of this work bears directly on the question of particle size effects, and is therefore beyond the scope of this article.

### 11.2. The Water-Gas Shift

This reaction has been extensively studied because of its industrial importance (Section 11.1); gold catalysts, especially when supported on reducible oxides, were shown by early work to have excellent activities [90], rates being reported at as low as 373 K [2]. It bears some formal similarity to CO oxidation, but CO has to be oxidised by water, a molecule more difficult to activate than O_2_. Various mechanisms have been proposed [2,91,92], a common feature being the role of the support, especially anion vacancies, in adsorbing water molecules, preliminary to its forming an OH group which then reacts with CO to give –COOH. Its dissociation proceeds as:

2 –COOH → 2CO_2_ + H_2_

The –COOH species has also been advanced as an intermediate in CO oxidation [20], and this has suggested that decomposition of HCOOH (formic/methanoic acid; see Section 11.3) would be a useful model reaction for testing catalysts for the Water-Gas Shift. The most active catalysts have gold particles smaller than 5 nm [2]. Au/TiO_2_(rutile) catalysts having particles between 1 and 7 nm show rates at 393 K that decline smoothly with increasing particle size [93], the ‘dominant active sites’ being corner atoms having CN <7. It is not however clear what happens at this site and whether everything takes place on it.

A surprising observation has been the effect of removing metallic particles from a catalyst by dissolution in aqueous NaCN; what remains was found to be as active as the starting catalyst [94]. It was at first thought that the residual gold was in the form of Au(III), and that this was the active form, but later work [95] has shown that it is reduced in the reaction. It is of course possible that Au^0^ atoms are dissolved from all particles, leaving only the charged species at the interface and periphery to become the source of activity in subsequent reactions. The low concentration and small size of what remains have frustrated attempts to characterize it [96], but regardless of this difficulty the work provides a further demonstration of the superior activity of very small gold particles.

### 11.3. Decomposition of Formic Acid

**Figure 12:**
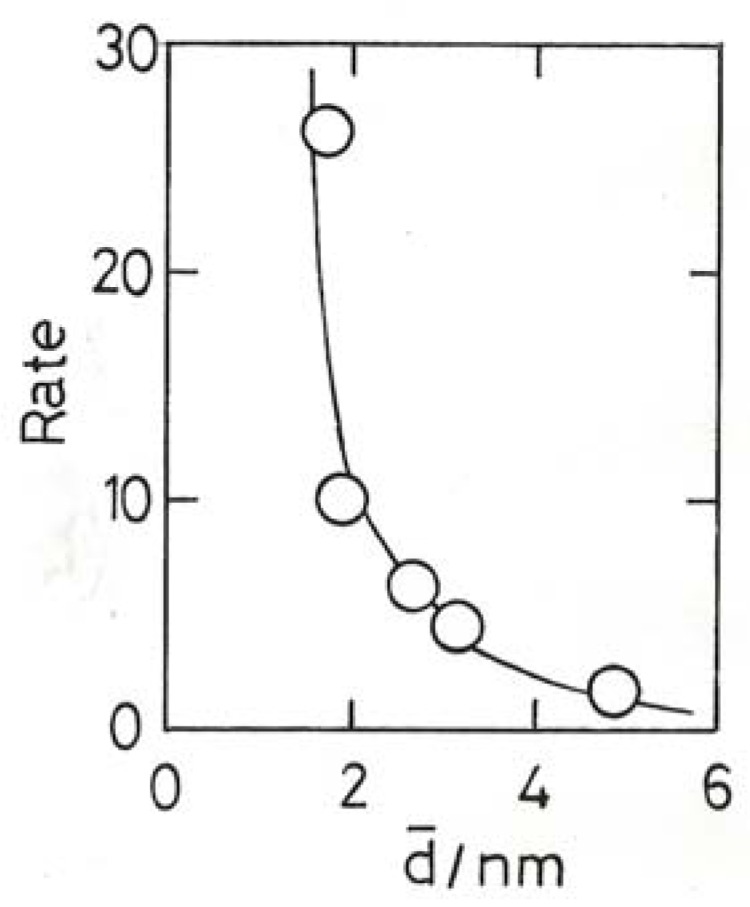
Rates of formic acid decomposition on Au colloids at ambient temperature [98].

Formic (methanoic) acid (HCOOH) decomposes on metallic catalysts to CO_2_ + H_2_ [63]. A recent study [97] has shown that NMR spectroscopy can be applied to formic acid adsorbed on metal colloids in aqueous suspension; the adsorbed state results when the formate ion HCOO^−^ is held symmetrically, the O atoms insulating the C atom form the metal electrons, so almost totally removing the complication of the Knight shift. Nevertheless the chemical shift of the C atom is responsive to the strength of binding of the ion and to the rate of decomposition. Rates were measured for colloidal gold particles between 1.7 and 4.9 nm (Figure 12); they start to increase rapidly below 2 nm. The rate-limiting step is probably:

HCOO^−^ → H* + CO_2_ + e^−^

The size effect presumably reflects the ease of finding a suitable site to accommodate the H atom, as well as the increased concentration of adsorbed formate ions as shown by the chemical shift (Figure 12).

### 11.4. Selective Oxidation of Propene to Methyloxirane

The efficient oxidation of propene to propene oxide (methyloxirane) by insertion of an O atom across the C=C bond has defied catalytic science, because of the greater ease of oxidation of the required product. Supported gold catalysts do however show very high selectivity, but generally only at low conversion [2]. The reaction requires the presence of H_2_, so that possible products include propane and water; the preferred support is TiO_2_ or a TiO_2_-containing SiO_2_ (e.g., TS-1) [98,99]. Small gold particles (<2 nm) appear to be essential, but with Au/TiO_2_ the smallest particles favour propane formation [100]. The reaction is thought to involve formation of –OOH species on the metal, and their migration onto the support, where the epoxidation then takes place. As with H_2_O_2_ synthesis (Section 11.1) it is unlikely that dissociation of O_2_ occurs, but if it does it will lead straight to water.

### 11.5. Electrocatalysis

The term applies to reactions taking place on the surface of a metal that is donating or accepting electric charge; a familiar example is the electrolysis of water to give H_2_ + O_2_, but of greater interest are the reverse processes whereby oxidation of H_2_ and reduction of O_2_ are coupled to generate a current of electrical energy in a fuel cell:

H_2_ → 2H^+^ + 2e^−^

O_2_ + 4e^−^ → 2H_2_O

The surface of massive gold has very limited electrocatalytic activity [101], most of which is due to atoms of low coordination number surrounding defects; this relates directly to the chemisorption of O_2_ on this type of surface. However small particles exhibit greater activity, and gold is one of the most effective metals for O_2_ reduction in basic media [102]. Marked particle size effects are shown by Au/C; 3 nm particles were 2.5 times more active than 7 nm particles, and gave 4e^-^ reduction to H_2_O, unlike the larger particles that gave only 2e^−^ reduction to H_2_O_2_. Similar behavior is shown in acidic media [103], and the size effect has been discussed theoretically [104]. A maximum in size dependence at 3 nm has also been observed in the electrocatalytic oxidation of CO [105]; perhaps here the non-metallic state is less effective than the metallic state because of the need to conduct charge. There are clear similarities with the gas-phase process, but electrochemists do not appear to converse with catalytic chemists.

## 12. Conclusions

The key to understanding the origin of the high catalytic activity of small gold particles, and of its variation with particle size, lies in establishing the probable nature of the active centre (not necessarily a single atom) through an analysis of the reaction kinetics, supplemented by spectroscopic measurements and theoretical calculations. The form of the reactants, including the strength of their adsorption, at the active centre then has to be explained in terms of the electronic structure of the participating catalyst species, which may include sites on the support as well as one or more gold atoms.

Defining the electronic structure of small gold particles, and specifically of those atoms likely to form the active centre, demands knowledge of the particle’s size and shape, the nature of species at the interface and periphery, the manner in which the particles is formed on the support, and the way in which the gold precursor has been reduced to the metal. Emphasis is however place in this review on the probable importance of very small particles (<3 nm) being in a non-metallic state, and on the effect that this may have on achieving an increased strength of reactant adsorption, as well as on the fraction of surface atoms able to act as adsorption sites. These variables may cause the particles to become either electron-rich or electron-poor, and atoms at the interface and periphery, and those in low coordination number positions, may have different electronic structures from other surface atoms, e.g., more *d*-level vacancies or fractional positive or negative charges; this may account for their often being held responsible for catalytic activity.
